# Past management affects success of current joint forestry management institutions in Tajikistan

**DOI:** 10.1007/s10668-018-0132-0

**Published:** 2018-03-15

**Authors:** L. Jamila Haider, Benjamin Neusel, Garry D. Peterson, Maja Schlüter

**Affiliations:** 1 Stockholm Resilience Centre, Stockholm University, Kräftriket 2B, 106 91 Stockholm, Sweden; 2 Deutsche Gesellschaft für Internationale Zusamenarbeit (GIZ), GIZ Office Tanzania, 65 Ali Hassan Mwinyi Road, Dar es Salaam, Tanzania

**Keywords:** Joint forestry management, Common pool resource management, Social–ecological, Resilience, Tajikistan

## Abstract

In the Pamir Mountains of Eastern Tajikistan, the clearance of mountain forests to provide fuelwood for an increasing population is a major source of environmental degradation. International development organisations have implemented joint forestry management institutions to help restore once-forested mountainous regions, but the success of these institutions has been highly variable. This study uses a multi-method approach, drawing on institutional analysis supported by Elinor Ostrom’s design principles and social–ecological system framework in combination with resilience thinking to help understand why some communities in Tajikistan manage their forests more sustainably than others. The application of the design principles provided helpful guidance for practitioners implementing joint forestry management. The social–ecological system analysis revealed both ‘history of use’ and ‘tenant density’ as positively associated with forest condition. However, we also identify limitations of snapshot social–ecological assessments. In particular, we illustrate the critical importance of considering historical legacy effects, such as externally imposed centralised governance regimes (that characterise many post-Soviet states) in attempts to understand current management practices. Our work shows how a more nuanced understanding of institutional change and inertia can be achieved by adopting a resilience approach to institutional analysis, focusing on the importance of reorganisation. Lessons learned from our analysis should be widely applicable to common pool resource management in other semi-arid forested landscapes as well as in regions with a strong centralised governance legacy.

## Introduction

In the Pamir Mountains of Eastern Tajikistan, the clearance of mountain forests for fuelwood is causing widespread soil erosion and negatively affecting a range of other important ecosystem services, such as the provision of timber building materials, conservation of wildlife habitat, recreational services and cultural values. Nearly all of Tajikistan’s agricultural lands have been reported as suffering from some degree of erosion, with winter pastures being particularly effected (Saigal 2003). This degradation has occurred primarily due to overgrazing and the felling of mountain forests for fuelwood and timber. These forests are a classical common pool resource, insofar as resource users cannot be excluded, while resources consumed by one user are no longer available to others (they are subtractable) (Ostrom 1990). This makes their management challenging as there will always be an incentive to individually use more than is beneficial for the community as a whole.

Many scholars (e.g. Berkes 1989; Leach et al. 1999; Agrawal 2001; Agrawal and Gupta 2005) have proposed that decentralised community-based management can achieve both conservation and human well-being goals in common pool resources by enhancing a community’s ability to self-organise. However, self-organisation is not always evident, especially in resource systems with a history of strong centralised governance (Barnes and Van Laerhoven 2013), which has led to non-governmental organisations taking a key role in the devolution of resource management (Shackleton et al. 2002; Blaikie 2006; Wright et al. 2016; Lund et al. 2018). In such instances, a common intervention strategy of both governmental and non-governmental development programmes has been to invest in building participatory governance mechanisms to help develop community-based natural resource management (Kumar 2002; Robinson et al. 2010). Joint forestry management (JFM) is one such approach that has been used to foster community-based management in many different parts of the world by transferring harvesting rights of forest products to rural people through contractual agreement with a relevant governing body (Robinson et al. 2010). Here we identify a tension between what scholars have observed in many cases: that resources users do self-organise, and the continuing practice of development organisations working to try to initiate collective action through community-based management planning. An interesting area of institutional scholarship is in contexts where practitioners aim to initiate collective action.

In the Pamir Mountains, joint forestry management has been introduced through a partnership between Deutsche Gesellschaft für Internationale Zusamenarbeit (GIZ) and the Tajik State Forestry Agency (which is commonly referred to by its Soviet name: *Leskhoz*) in an attempt to tackle problems of widespread forest loss and soil erosion. Yet the success and adoption of joint forestry management have varied across the region (Mislimshoeva et al. 2016). We examine the factors behind this variation using a novel multi-method approach that combines social–ecological institutional analysis and resilience thinking. Our institutional analysis first draws on the social–ecological systems framework (henceforth SES framework) (Ostrom 2007, 2009) to identify and analyse variables that may help explain differences in the outcome of JFM application, and secondly on Ostrom’s design principles for robust property rights institutions (Ostrom 1990) to assess how conducive the actual design of joint forestry management in Tajikistan is to success. Both of these frameworks were developed by Elinor Ostrom through the analysis of conditions for successful self-governance across a large number of common pool resource case studies. In our Tajik case, property rights have been formally allocated to tenants under the JFM programme. Nevertheless, the de facto open access nature of forests in Tajikistan, even under joint forestry management, lends themselves to a common pool resource framework. Resilience thinking is used as a complementary social–ecological approach to identify relevant slow variables affecting the present-day outcome in forestry management.

This research seeks to inform both practice and theory by: (a) improving our understanding of the conditions under which joint forestry management can be successful in the Pamir Mountains; (b) providing concrete suggestions for how to improve the development of participatory resource management as pathway for improving the well-being of local communities; and (c) suggesting opportunities to better account for factors leading to successful management across space and time in complex social–ecological systems with strong historical legacy effects from a centralised government. Our study aims to address various aspects of the impacts of decentralisation on: forest quality, institutional empowerment, and the importance of historical and contextual factors that influence common pool resource management today (thereby addressing a gap in forestry decentralisation literature identified by Lund et al. 2018).

The paper proceeds as follows: we first describe forestry management and its history in the Pamir Mountains, followed by a description of our multiple method approach to understanding institutional variation and its effects on natural resource management in a dynamic historical context. We then present results on how independent variables identified in the SES framework (such as history of use and group size) effect forest condition (planned harvest) from survey and interview data collected from all JFM communities in the Pamirs (a sample of 25). The quantitative results are complemented with an in-depth comparison of three communities with varying JFM success. We conclude by discussing the implications of our findings for forestry management in the Pamirs and beyond.

## Background

### Introduction to the case study: The Pamir Mountains, Tajikistan

Tajikistan’s transition from a command-and-control governance system to a market-based economy after independence from the Soviet Union in 1991 was turbulent (Giffen et al. 2005). Its economy, based primarily on cotton and aluminium exports, was vulnerable to external price fluctuations, and the economy could not create enough jobs for all the labourers coming from state-run farms and factories. The country precipitated into civil war (1992–1997) between the Tajik and Pamiri people, further contributing to economic stagnation. Since 2000, the GDP has expanded by almost 10 per cent, but a substantial part of the overall expansion is comprised of remittances from migrant workers in Russia and other Central Asian states (UN Data 2013). Still, 41 per cent of the population lives under the poverty line of $2.15/day, with 75 per cent of the poor living in rural areas (World Bank 2009). The legacy effects of the transition and the civil war still have profound impacts on the country, with violent uprisings in 2012 and 2014 continuing to destabilise the region (‘Tajikistan Clashes’ 2012).

**Fig. 1:**
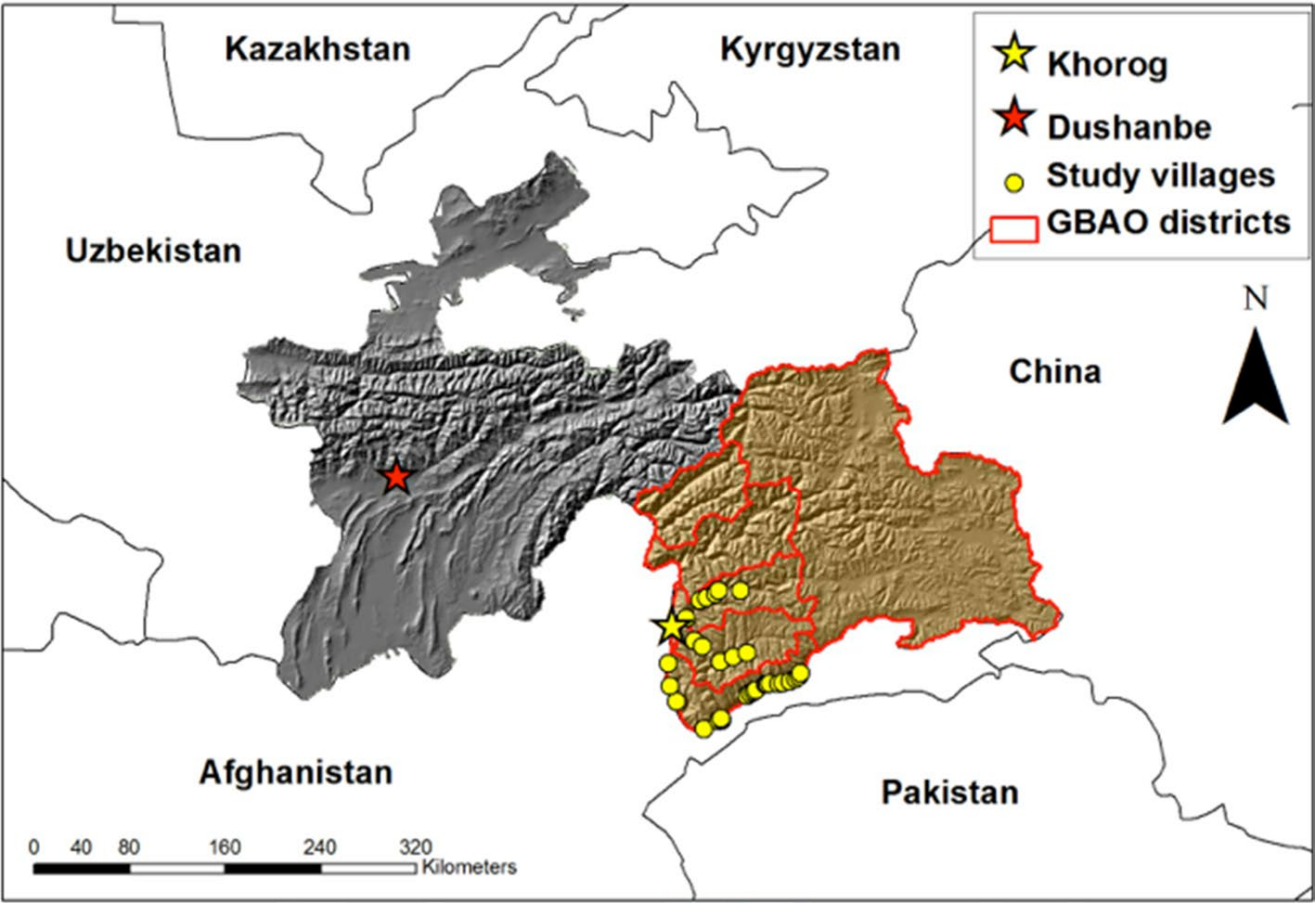
Map of Tajikistan (coloured), with Gorno-Badakhshan Autonomous Oblast, or the Pamirs in light brown, and the rest of Tajikistan in grey. Yellow points indicate villages where JFM has been implemented and surveyed for this study. The topographic map demonstrates how villages are located on valley floors

Gorno-Badakhshan Autonomous Oblast, often simply referred to as the Pamirs, is Tajikistan’s largest province, constituting 64,200 km^2^, and is the poorest region of the country (Fig. 1). It is sparsely inhabited with a population of only 220,000 people (GIZ 2012). A strategically important buffer for the Soviet Union to China and Western influences to the east and south, the Tajik Pamirs benefitted from decades of Soviet modernisation through swift technological and economic development. As a result of large imports of fuel, fodder and food, its population quadrupled during the Soviet era (Breu et al. 2005). During the Soviet era, the central government recognised the inherent fuel limitations of the area and the ecological risk of desertification and initiated afforestation campaigns. Strictly regulated quotas on wood harvest were also imposed, and the fuel needs of a growing population were met by importing gas and petrol and supplementary wood sources. This liberal inflow of fuel resources ended abruptly in 1992, when Tajikistan fell into civil war. The Pamiri people rebelled against the central government in Dushanbe and became almost completely isolated from the rest of the world for 5 years. The absolute cut-off from imports, coupled with the collapse of the Soviet command-and-control governance systems, forced most people to turn to the forest for fuel, resulting in severe ecological degradation of forests within a decade (Herbers 2001; GIZ 2012). In the shift back to a subsistence-oriented economy, small fuelwood has once again become the primary source of fuel after decades of gas and petrol imports from Russia.

### Forest derived ecosystem services

Forests occur only in the western Pamirs, where the average altitude is 4060 m, precipitation is less than 200 mm and only 0.4 per cent of the land is arable (Breu and Hurni 2003; Hergarten 2004). The forests of the western Pamirs are primarily riparian as limited water availability restricts most tree growth to alluvial plains. They are commonly referred to as Tugai forests throughout Central Asia. Tugai forests are characterised by a mix of fast-growing tree species: poplar (*Populus spec.*), willow (*Salix spec*.) and shrubs such as seabuckthorn (*Hippophae rhamnoides*), and salt cedar (*Tamarix spec.* indicating desertification*)* and occur up to altitudes of 3400 m (Kirchoff and Fabian 2010). Despite forests covering less than 2% of the Pamirs, they remain an important source of rural livelihoods, providing fuelwood, grazing land and a source of collected fodder. The mean annual fuelwood consumption per household is estimated to be 3–4 m^3^. As the demand is much higher at approximately 20 m^3^ (Kirchoff and Fabian 2010), a large proportion of this demand is supplemented with an energy mix of dung, electricity and coal. The need for fuelwood and grazing pressure in the forest are the main drivers of forest degradation.

The forests are also of critical importance for controlling widespread soil erosion. Steady winds are a characteristic feature of many of the river basins resulting in the deposition of sand on fields and the denudation of fertile soil. Vegetation cover, however scarce, plays an important role in mitigating these processes (GIZ 2012). Where forests have been cut, sand dunes accumulate and threaten arable land and villages. Forests also play an important role in the rich culture of narrative, poetry and music of the Pamiri people that is closely intertwined with the landscapes they inhabit (Kassam 2010; van Oudenhoven and Haider 2012). Timber is used to construct the *roetz* (roof window) of the Pamir house, which represents four Zoroastrian elements, and poplar timber is used to construct the five main pillars of the house, representing the Prophet Muhammad, his daughter Fatima and son-in-law Ali, and their two children (Bliss 2006). Every traditional house in the Pamirs is constructed this way, and the structure is known to be resistant to earthquakes. Wild juniper twigs are used for the blessing of the home each New Year, and the wood of fruit trees has many specific traditional uses, such as utensils and bowls for ceremonial dishes (van Oudenhoven and Haider 2015).

### Forestry management in the Pamirs today

In 2009, the *Leskhoz* began implementation of JFM with support from the development organisation Deutsche Gesellschaft für Internationale Zusamenarbeit (GIZ), as a means to regain control over forest resources. After the collapse of the Soviet Union, *Leskhoz* remained the management body only in name, with its staff reduced to a handful of individuals at the local level, operating without computers, without vehicles and often without electricity. In the context of unsustainable forest exploitation and the institutional down-sizing and mismanagement of the *Leskhoz*, the goals of JFM defined jointly by GIZ and the *Leskhoz* were twofold: to reduce forest degradation and second, to ensure an equitable and regulated distribution of the benefits provided by the forest to forest user households.

**Fig. 2:**
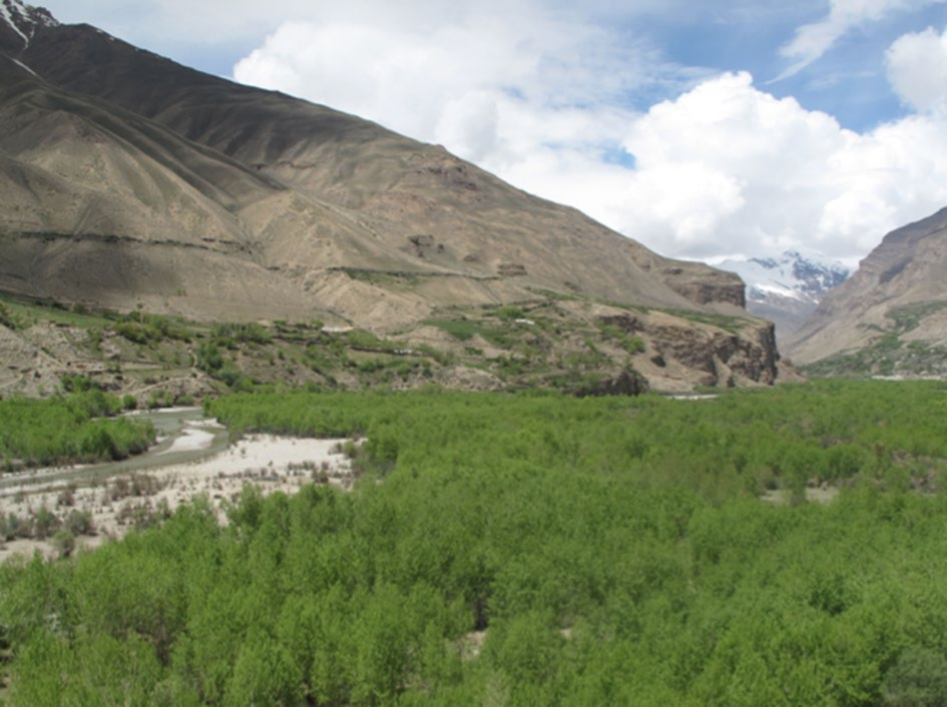
Forest in Roshtkala valley, dominated by *Populus* and *Salix* understory. In the Pamirs, this forest is on the higher end of forest productivity

The JFM approach in Tajikistan is based on a detailed use contract whereby *Leszkhoz* delegates 20 years of use rights for forest access to local people who become tenants. *Leskhoz* supports the tenants in developing an annual plan of forest management activities, including planned harvest and maintenance and monitors its implementation. The forest users have the responsibility of maintaining, developing and protecting their forest plot, and in return receive a share of the forest products, allocating 70% to the forest tenants and 30% to the *Leskhoz*. Approximately 2000 ha of state forestland in three districts of Gorno-Badakhshan Autonomous Oblast (GBAO) is currently under JFM management, which makes up 20% of actual forest cover in GBAO, involving about 350 households in 40 communities (Fig. 2 offers an example of a typical forest in the Pamirs).

## Methods

### Multi-method research approach: institutional analysis and resilience thinking

**Fig. 3:**
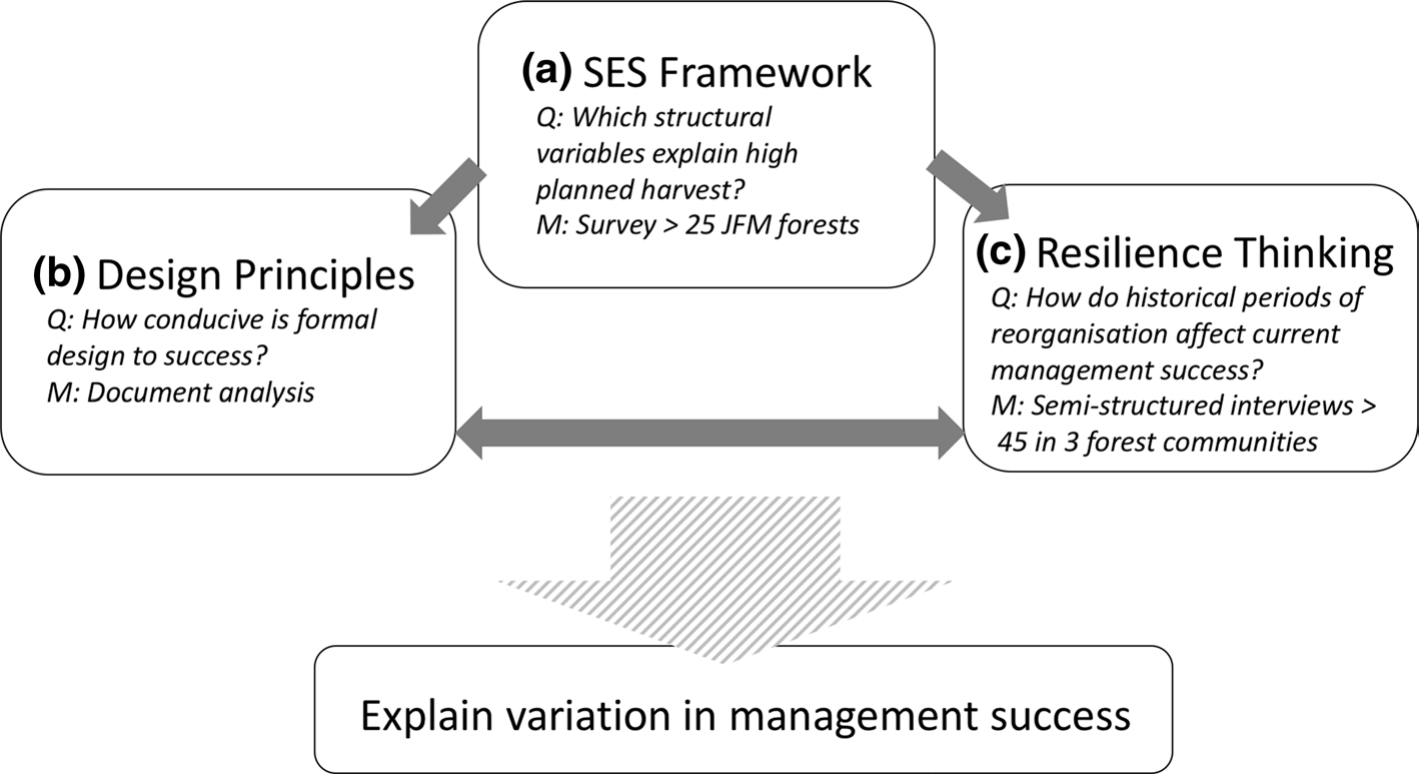
Description of research steps to understand variation in successful forest management

We use a three-tier method to assess the institutional design of the joint forestry management programme, helping to understand variation in management success and the way in which institutional dynamics over time have influenced management outcomes (Fig. 3). Forests are managed in three different ways in the Pamirs: (1) the majority of forests are JFM (35 forests); (2) forests which have their own governance structure (3 forests); and (3) *Leskhoz* forests which remain under jurisdiction of the *Leskhoz* (5 forests). We are only concerned with JFM forests in this study.

The SES framework is the central analytic method, with more descriptive (design principles) and qualitative methods (resilience approach) used to elicit the cross-scale dynamics we hypothesise to be present in the institutional variation of joint forestry management. The SES framework (Fig. 3a) was used to select relevant variables in explaining variation in planned harvest, testing through focus group surveys in each of the case study communities. In total, the survey was carried out in 35 JFM forests. The unit of analysis is JFM forest rather than village, since in a few cases, two villages share a forest. The survey was carried out by GIZ staff, called ‘mobilisers’ who have long-term relationships with the forest tenants. In order to conduct the survey, the responsible mobiliser followed the survey protocol (Appendix A) and asked the survey questions to a focus group of all forest tenants who were able to attend the meeting at this time. Dispute or conflict around answers within the group were recorded and used to elaborate on the results of the paper.

The experience of conducing the survey in focus groups of JFM forests revealed that many design principles were not being met in practice, which invoked the complementary document analysis (Fig. 3b) to answer how conducive the formal design of JFM was to success. Results from the survey helped identify which forests would be interesting cases in which to do more in-depth analysis (Fig. 3c) for the purpose of understanding how historical dynamics have shaped current management success. Three cases were selected to represent a range of successful JFM adoption. We use resilience as a conceptual framing to incorporate temporal and multi-scale dynamics into the analysis, complementing the SES framework. The following paragraphs describe each of these methods in greater detail.

#### SES framework: analysis of factors explaining variation of planned harvest

The SES framework is a multilevel, nested collection of variables that have proven to be relevant for explaining the emergence and success of local common pool resource management institutions (Ostrom 2007, 2009). The SES framework is meant to provide a common framework for social and natural scientists to think collectively about which factors in a given context contribute to more or less sustainable management of resources (Ostrom 2009). The framework breaks down a social ecological system into six core subsystems or first-tier variables: resource systems, resource units, governance systems, users, and interaction and outcomes, each containing second-tier variables which interact leading to specific outcomes. The selection of these categories of variables is based on three decades of empirical work studying common pool resources (Ostrom 1990; Gibson et al. 2005) and is being continuously adapted and expanded by scholars (see Epstein et al. 2013; McGinnis and Ostrom 2014; Leslie et al. 2015; Taggart-Hodge and Schoon 2016; Tyson 2017). The second-tier variables can be further unpacked to third- and fourth-tier variables and so on, to account for the specific variables of a case. Selection of relevant variables for a specific case and research question is an important and difficult step for an institutional analyst, and documenting the rationale for variable selection to assess forestry management is one important contribution we make with this paper to scholarship on common pool resource management (see Table 1).

**Table 1:** Initial set of variables as hypothesised to explain forest condition in GBAO, Tajikistan (codes correspond to SES framework variables (Ostrom 2009)

First-tier variable	Second-tier variable selected	Operationalised:	Hypothesis
Resource system (RS)—forest	Size of resource system (RS3)	Forest area (ha)	Very large territories are difficult to self-organise given high costs of defining boundaries and monitoring, while very small forests do not generate substantial flows of products (Chhatre and Agrawal, 2008; Baland and Platteau 1996)
	Productivity of system* (RS5)	Expert opinion on planned harvest	*Outcome of interest/dependent variable
	Location (RS9)	District/valley	Differences between districts capture an important dimension of variability based on place-based ethnic and language differences. Districts are defined by valleys, each of which have different types of users/ethnic groups
Resource unit (RU)—fuelwood/timber/edible biomass	Interaction among resource units (RU3)	Grazing intensity (forests are used for timber resources and grazing of livestock)	Grazing intensity was identified as a major determinant of forest condition, as is therefore tested under interaction between units. Forests in the Pamirs which are used primarily grazing are treated more like open-access pastures rather than managed forest plots. Intensity of grazing may have a negative relationship to value of the forest for timber resources (Agrawal and Chhatre 2006)
Governance system (GS)—JFM	Nongovernment organisations (GS2)	The presence of self-organised community-based groups (not JFM)	The existence and strength of parallel formal governance structures for resource management (such as water associations and house building collectives) indicate collective action potential and an interest in natural resource management beyond the scope of the government programme and will therefore maybe have a stronger affinity to organise for natural resource management
	Operational rules (GS5)	Existence of operational rules and strength of rule compliance	Forest condition is likely to be higher if operational rules are understood and followed (Nagendra 2007)
	Monitoring and sanctioning (GS8)	The presence of monitoring	Regular monitoring and sanctioning will lead to the maintenance of higher forest condition (Gibson et al. 2005; Cox et al. 2010).
		Sanctioning (user knowledge of penalties)	Forest condition is positively associated with effective sanctioning, i.e. if users are aware of penalties (Chhatre and Agrawal 2008; Cox et al. 2010).
Users (U)—tenants	Number of users (U1)	Tenant density	The effect of group size on self-organisation depends on the other variables in the social–ecological system and the type of management tasks envisioned. Small group sizes may have high transaction costs for organisation, while large group sizes have the same issue with regards to agreeing on changes (Agrawal and Yadama 1997; Nagendra 2007). Probability of degradation increases with increasing proportion of firewood needs supplied from forest (Chhatre and Agrawal 2008). Given variable forest size, tenant density used a more relevant measure of group size
	History of use (U3)	Forest age (pre-Soviet or Soviet)	Districts with longer history of community-based forest management will have higher productivity (Lynch et al. 1995; Brosius et al. 1998).
	Leadership (U5)	The presence of leader (scale: absence, weak, moderate, strong leadership)	A strong leadership figure in forestry management in the community will be positively associated to subsistence value (Baland and Platteau 1996)
Interactions–outcomes	Conflicts among users (I4 and I3)	The presence of conflict and resolution arena	The presence of conflict (yes/no) will be assessed qualitatively to understand how the community adapted to and dealt with conflict. Villages with conflict resolution arenas would be more likely to self-organise (Ostrom, 2009)
	Self-organising activities (I7)	The presence of self-organising activities	Self-organisation outside of scope of JFM are thought to be positively associated with forest condition

More information on operationalisation is found in “Appendix 1” survey

In order to select second-tier variables from the framework that were relevant, attainable and useful for explaining differences in planned harvest of Pamiri forests as the ecological outcome of interest, we reviewed synthesis studies on the governance of forestry resources (Gibson et al. 2005; Chhatre and Agrawal 2008, Coleman 2011), the International Forestry, Resources and Institutions (IFRI) Database (IFRI 2008) and relevant case studies from other high altitude areas in India and Nepal (Agrawal and Chhatre 2006; Nagendra 2007). Based on this review, we selected ‘planned harvest’ as an outcome variable to act as a proxy for forest condition.

Planned harvest (a social–ecological variable) is an expert assessment by local foresters measured annually as part of management planning (we use 2012 data). Forestry officials from *Leskhoz* assess the forest each year for planned harvest (for timber and firewood) and establish the plans accordingly. This provided us with a reasonably consistent estimate of forest condition. The planned harvest measurement is an estimate, based on a reference forest. A ‘good’ forest is used a reference forest, characterised by a dense forest with little damage from grazing. Forest officials compare other forests to this reference forest by estimating (a) how the density of the forest compared to the reference forest, (b) how many new trees had been planted, and (c) whether or not grazing was a problem. No ecological data of forest condition exists across the case study forests, and therefore planned harvest is an appropriate indicator, taking both social factors (harvest need) and ecological factors (maximum sustainable yield) into account.

Variables were further selected through expert consultation: the field team of GIZ identified SES framework variables to be excluded which they thought were (a) not achievable given temporal and spatial data collection limitations, or (b) contextually irrelevant, or (c) confounding. Table 1 shows the variables that were selected for our analysis. We develop a set of hypotheses based on both theory and empirical observation to test which variables are associated with sustainable forest management (Table 1). Variables we considered to be relevant but did not test (due to a lack of variation) include: boundaries (all JFM forests are clearly defined with fences), proximity to road (all forests are along a road), collective choice rules (*Leskhoz* defines rules), constitutional rules (national-level *Leskhoz* defines rules), illegal use (too difficult to assess) and importance of resource (subsistence use for all).

The data for the selected SES framework variables were collected from previous assessments and through a field survey carried out in the same sites where forest condition was assessed by local officials. The surveys (*N* = 25 JFM forests) were conducted in focus groups of forest users in all the JFM forests in the western Pamir region (with groups ranging from 2 to 18 tenants), with some villages being grouped together for the focus group when they had two or less tenants available for the survey. Even in these cases, a separate survey was completed for each JFM forest. Responses were cross-checked with non-tenants for qualitative supplementary information to ensure the tenants’ responses were not biased towards a positive representation of the JFM programme (survey found in “Appendix 1”).

We examined the relationship between hypothesised explanatory variables (see Table 1) and estimates of forest productivity. We first examined the correlation among variables and then used hierarchical partitioning to identify variables that had a significant independent effect on estimated forest productivity. Hierarchical partitioning is a statistical method that analyses all possible models in a multiple regression to identify the contribution of each variable to the total variance, both independently and in conjunction with the other variables, to infer the impact of each variable (MacNally 2002). We conducted this analysis using the R statistical environment (R Core Team 2013) and the package ‘hier.part’ (MacNally and Walsh 2004).

#### Design principles: institutional analysis of joint forestry management rules

Elinor Ostrom developed a well-known set of institutional design principles of community natural resource management based upon her extensive empirical research (Ostrom 1990). Here we use the extended list of design principles (Cox et al. 2010) to assess the potential for success of JFM given the formal rules in place under the auspices of JFM in the Pamirs (Fig. 3). The principles describe conditions under which collective action for sustainable resource use is more likely to be achieved (Ostrom 1990; Ostrom et al. 1999; Cox et al. 2010). They can be used to assess the potential of a given institutional design to lead to successful self-governance; however, they cannot explain variation in management success in common pool resource institutions that come about from different real-world contexts (the rules-in-use) of similarly designed common pool resource institutions. The design principles were intended for community-scale resources management and are therefore appropriate for this study, but it should be noted that modifications to the principles for larger-scale common pool resource management have been suggested by Lacroix and Richards (2015). Data on the design and implementation of JFM by GIZ were collected through participatory observation and document review (project documents, progress reports, monitoring reports).

#### Resilience approach: assessing temporal and cross-scale determinants of the success of joint forestry management

To analyse how collective action can be initiated by an external actor (such as GIZ), we complement the snapshot provided by institutional analysis with an analysis of the cross-scale interactions of biophysical systems with their users and governance regimes over time using a resilience lens. Resilience as we use it here is the capacity of a social ecological system to continually change and adapt yet remain within critical thresholds to allow for development along its current trajectory (Folke et al. 2010, 2016). We draw on two particular aspects of resilience thinking to help understand the dynamics of institutional change: the adaptive cycle and slow variables. The adaptive cycle metaphor (Gunderson and Holling 2002) is used in this study to classify periods of systemic change and reorganisation and identify slow social–ecological changes that shape current patterns of forest condition and governance. The reorganisation phase is when novel changes can emerge, and windows of opportunity for change open up (Biggs et al. 2010), and is a central concept of resilience thinking (Folke 2006). It therefore also seems to be an appropriate focal phase for institutional scholars or practitioners interested in initiating collective action in common pool resource management. If our research had a stronger policy objective, we could have also drawn on punctuated equilibrium theory (True et al. 2007) to explain the path dependency of institutional characteristics following a period of crisis. Path-dependency theory (Mahoney 2000) could be employed as an additional method to analyse deterministic contingent events which have led to certain institutional outcomes. We find the adaptive cycle to be an appropriate starting point to consider which historical social and ecological factors matter (Darnhofer et al. 2016).

The cross-scale dynamics addressed here included temporal dynamics (past with present, and slow and fast variables), as well as various organisational scales (national and local scales). We looked for slow variables (sensu Walker et al. 2012) such as increase in grazing pressure and demographic change to see how the faster dependent variable under study (planned harvest) responded to variation of key variables which changed across temporal scales (in this case collapse of the Soviet Union and end of the civil war).

Forty-five interviews were conducted in three comparative case study villages in order to understand cross-scale interactions, particularly historical factors. The selection of villages was based on expert opinion to represent a range of outcomes: village A refused JFM; village B tried to implement JFM, but with limited success; village C successfully adopted JFM. The interviews were conducted in a semi-guided style (Patton 2002) where topics were specified in advance but were reworded as necessary throughout the interview. Purposeful sampling with maximum variation was used as the sampling strategy for the semi-structured interviews (Miles and Huberman 1994, Creswell 2007) in order to choose participants who were best able to provide information on changes in forest condition over time (i.e. elderly forest users; see “Appendix 2”). Transect walks were used to engage tenants in describing general resource use patterns.

We analysed resilience dynamics by identifying temporal periods that corresponded to different phases in the adaptive cycle—in particular the phases of conservation, crisis and reorganisation. We organised interview responses based on phases of forestry management and slow changes in social and ecological factors related to the contrasting forest management outcomes in the three villages by exogenous (e.g. property rights) and endogenous (e.g. leadership, collective action, need for provisioning services) drivers.

## Results

### Design principles for collective action

The evaluation of Ostrom’s institutional design principles revealed that four design principles are met (clearly defined boundaries, proportional equivalence between benefits and costs, graduated sanctions, minimal recognition of rights to organise); two are only partially met (monitoring, nested enterprises), and two have not been met (collective choice arrangements, conflict resolution mechanisms) (Table 2). Column 2 describes whether the design principle exists in formal written records, and column 3 assesses the outcome in practice.

**Table 2:** Summary of achievement of design principles (adapted from Cox et al. 2010)

Design principle	Included in formal design of JFM? (data based on document analysis)	Outcome in practice (data based on survey results)
1A. Clearly defined boundaries (individuals who have rights)	Yes, tenants are officially granted lease.	Legal tenants are known to community members, but selection process is less than transparent and tenants not always accepted by all
1B. Clearly defined boundaries (of resource)	Yes, forest plots delineated by fences (Kirchoff and Fabian 2010)	All JFM forests are fenced
2A. Proportional equivalence between benefits and costs: rules restricting time, place, technology and quantity of resource units	Yes, planned harvest and management plan agreed by forestry expert, government and tenants	60% of user groups responded that they had adequate fuelwood sufficiency
2B. Proportional equivalence between benefits and costs: benefits obtained by users are proportional to the amount of inputs	Flexible proportion: ranging between 30 and 70% for forest user/*Leskhoz* ratio depending on length of contract	General agreement with proportion. Rumours of corruption and misuse (over harvesting by *Leskhoz*) in some cases
3. Collective choice arrangements	No, *Leskhoz* does not recognise rights of communities to modify operational rules	Two respondent communities felt they had some authority to affect rules
4A. Monitoring: Monitors are present and actively audit CPR conditions	Yes, *Leskhoz* and GIZ staff monitor success of management plan	Monitoring takes place regularly
4B. Monitoring: Monitors are accountable to or are the appropriators	At time of data collection, tenants did not have primary monitoring responsibility	However, 53% of the user communities perceived monitoring to be the primary responsibility of the NGO. Only 17% of the respondents felt that tenants had any responsibility at all
5. Graduated sanctions	Yes, fines per m^3^ x increasing factor of monthly salary for firewood, timber, grazing (SFA 2012)	Users know that sanctions exist, however, 13 of 25 communities were unaware of the accurate values of the penalties
6. Conflict resolution mechanisms	No (but with plan that forest committees would support this)	60% of user groups reported conflict around livestock grazing in the forest and no adequate arena in which to resolve disputes
7. Minimal recognition of rights to organise	Yes, committees may be formed within village organisation (VO) structure	8 committees
8. Nested enterprises	No	Not necessary for small-scale resource management

### Social ecological system variables explaining forest condition

**Fig. 4:**
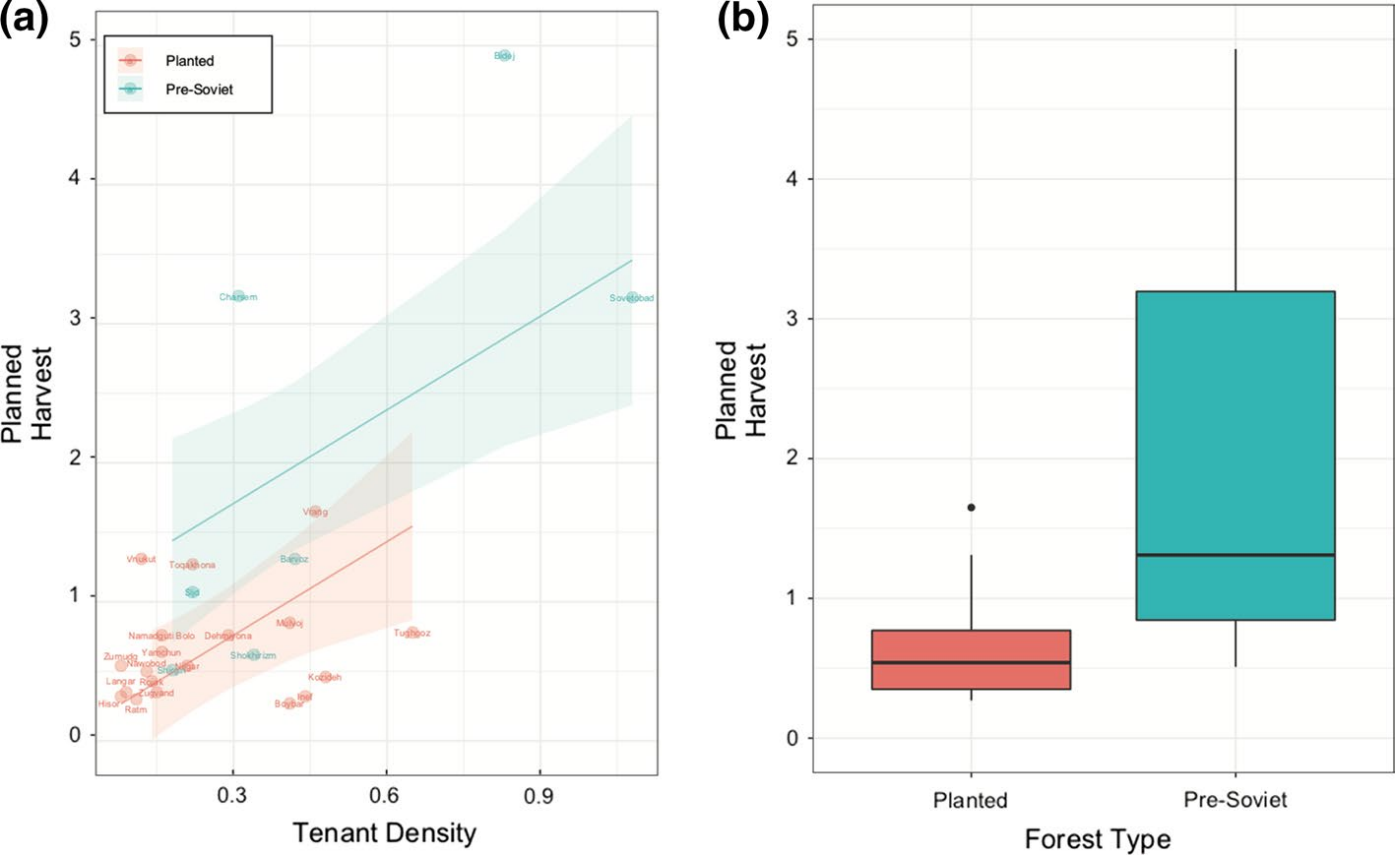
**a** Relationship between tenant density and planned harvest is different between pre-Soviet- and Soviet-planted forests. **b** Productivity of pre-Soviet forests is much higher than Soviet-planted forests

Significant positive associations were found between planned harvest and tenant density (adj *R*^2^ = 0.414, *p* < 0.001). However, we found no strong relationship between planned harvest and any of the other SES variables. Examining the relationship between villages and planned harvest revealed substantial differences between villages which had forests established before the formation of the Soviet Union and those planted during the Soviet Union (Fig. 4; adj *R*^2^ = 0.334, *p* = 0.0012). Although the SES variables are correlated with one another, the hierarchical partitioning analysis revealed that tenant density and forest type were the only variables to have a significant independent effect (Table 3). A multiple regression showed that planned harvest was positively related to tenant density for both planted and pre-Soviet forests, although harvests were much greater in pre-Soviet forests (adj *R*^2^ = 0.5226, *p* < 0.0001) (Fig. 4).

**Table 3:** Regression analysis of variables affecting forest condition as estimated by planned harvest (significant results in bold)

Variables	Units	Description	Regression	Significance *p*
*Dependent*				
Planned harvest	m^3^/ha	Planned harvest 2012 assessment by forester per plot area		
*Independent*				
Tenant density	tenants/ha	Number of tenants per plot area	adj *R*^2^ = 0.414	**<0.001**
Leadership		Existence and strength of present leader	adj *R*^2^ = − 0.042	0.998
Forest history	Planted/pre-Soviet	Soviet-planted forest versus pre-Soviet plantation	adj *R*^2^ = 0.334	**0.0012**
Penalties and fines	Proportion of penalties known	User knowledge of penalties and fines	adj *R*^2^ = − 0.041	.9428
Tenant density and forest history	Planted/pre-Soviet	Soviet-planted forest versus pre-Soviet plantation	adj *R*^2^ = 0.5226	**<0.0001**

Forests established before the Soviet era were likely to be in better condition than forests established during the Soviet era. When we look at tenant density and forest history in combination (Fig. 4), we see that a lot of the observed variability in planned harvest is linked to the history of forest use and that three villages (Charsem, Bidej and Sovetobad) have much more productive forests than others. These are all villages that have pre-Soviet forests. Overall, forest condition improved with tenant density; however, there is only a relationship between tenant density and planned harvest in the Soviet-planted forests. The mean value of planned harvest in these pre-Soviet forests is higher than that of Soviet-planted forests (1.0 vs. 0.73 m^3^/ha/year) (see, for example, Fig. 2, which is pre-Soviet).

Leadership, penalties and fines were all identified as important variables explaining forest condition in the qualitative interviews, but were not found to be statistically significant.

### Applying dynamics from resilience thinking to institutional analysis

The three villages selected for in-depth interviews contrasted primarily in their acceptance of JFM as a management approach. Village A chose not to adopt the JFM approach because the forest is their primary winter grazing land. The head of the regional forestry office allows 23 households whose property backs directly onto the forest to cultivate crops and even establish small buildings, despite this being strictly against the forest code, in order for them to actively manage the forest. The rest of the forest is open access and nearly completely deforested with only a few remaining poplar trees and seabuckthorn and is effectively used as pastureland. Livestock herding is the main source of livelihood (focus group, FG2) and is dependent on the forest: ‘The forest is our only opportunity for grazing’ [non-tenant 1, non-tenant 2 (see “Appendix 2”)]. Many of the poorer people in the community said that they would want JFM because it would give them land to graze, but only if it was evenly distributed among all households—which would mean that each tenant would be left with less than 1 ha of forest. The current wealthier plot owners refused this proposal, since they would lose land they have depended on for over 25 years, and the livestock would be unlikely to survive the winter without access to the forest.

Situated in the lower Ishkashim valley, village B agreed to JFM as a management approach but failed to implement it successfully, regularly failing to meet management plans, resulting in eight contract cancellations in 2012 by *Leszkhoz*. The cancellation of these eight contracts exacerbated mistrust between the government and community members (non-tenant 4). Due to the difficulty in managing the large number of uncooperative forest users, the *Leskhoz* has suggested paying one person in a full-time position to manage the forest, but nobody has accepted this offer, stating that the management of the forest is an impossible task (tenant T3). The forest was planted in 1957 by the *Kolkhoz* (Soviet collective farm) when the population of the village was 15 households, compared to 66 households today. During this time, a very strict local forest official managed the forest and it was in good condition, because of his strict, often physically abusive, enforcement of punishing any rule breakers (Forest Official 1). Another reason for the diminished responsibility community members feel towards management of the forest is a local military base, which shares a 400-m border with the forest, with soldiers allegedly stealing a lot of fuelwood at night as the base has no electricity or source of heating (tenant 2).

**Fig. 5:**
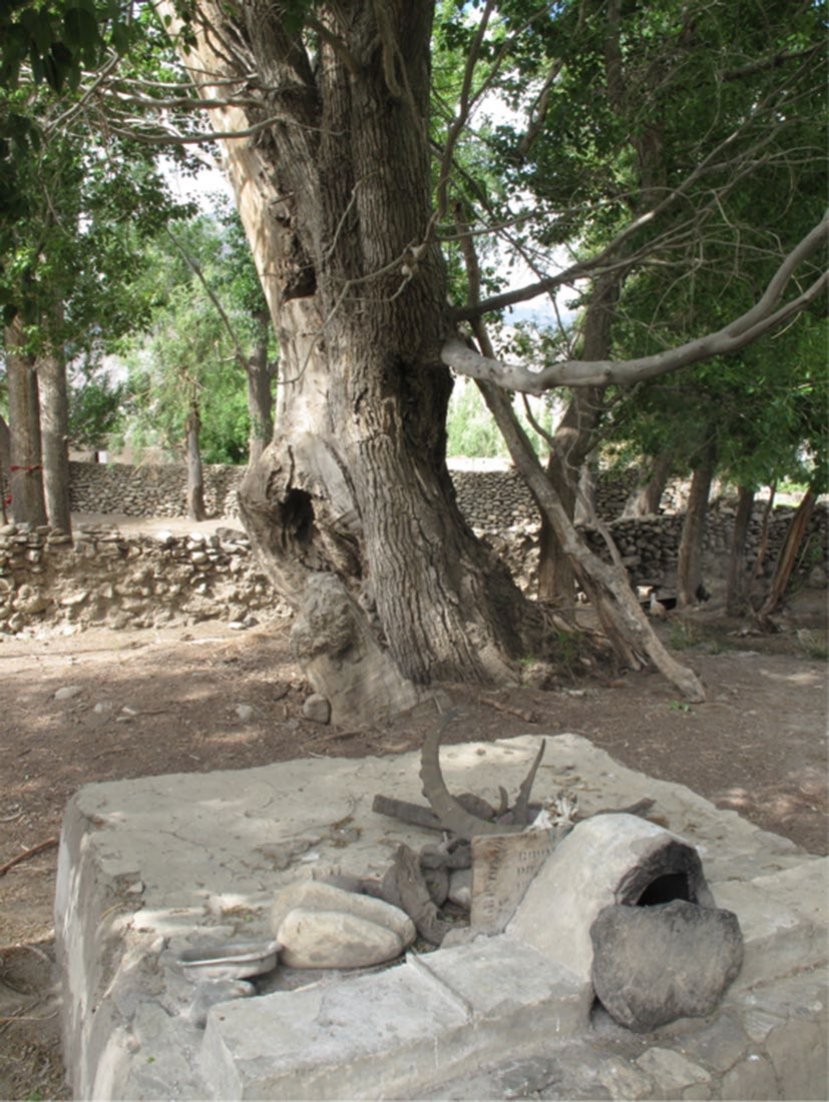
Shrine in village C, which consists of four planted poplar trees enclosed with offerings of goat trophies. These are holy trees, where people practise khudoi*—‘*in the name of God’, a practice for communal prayer after an accident or illness. The family of the inflicted makes a fire using dried fallen branches and cooks the traditional meal, *Boj,* which they share with the community. While the holy site could not be considered to have the potential conservation value of a sacred grove, it holds symbolic significance with regard to the importance people attribute to trees (Photo: L.J. Haider 2012)

Village C readily adopted JFM and regularly meets its annual planed harvest targets. The village is also in Ishkashim district, but is located in the upper reaches of the valley, in the Wakhan corridor, which is inhabited by Wakhi people. The Wakhi are a small ethnic group who speak their own language (Wakhi) and practise combined mountain agriculture (Kreutzmann 2003). Initiated by the village head, seven men formed a forestry management group during the civil war to halt deforestation. These seven men still play an active role in forest monitoring, maintenance and conflict resolution today. In addition to strong leadership, village C places notable spiritual value on trees, much more so than other villages. Two sacred groves exist. One is the location of many religious ceremonies, while the other hosts an Islamic shrine that was built in honour of Hazarati Ali, who is thought to have brought Islam to the Pamirs in the sixth century (non-tenant 5). Every village in the Wakhan has shrines around groups of trees (Fig. 5).

**Fig. 6:**
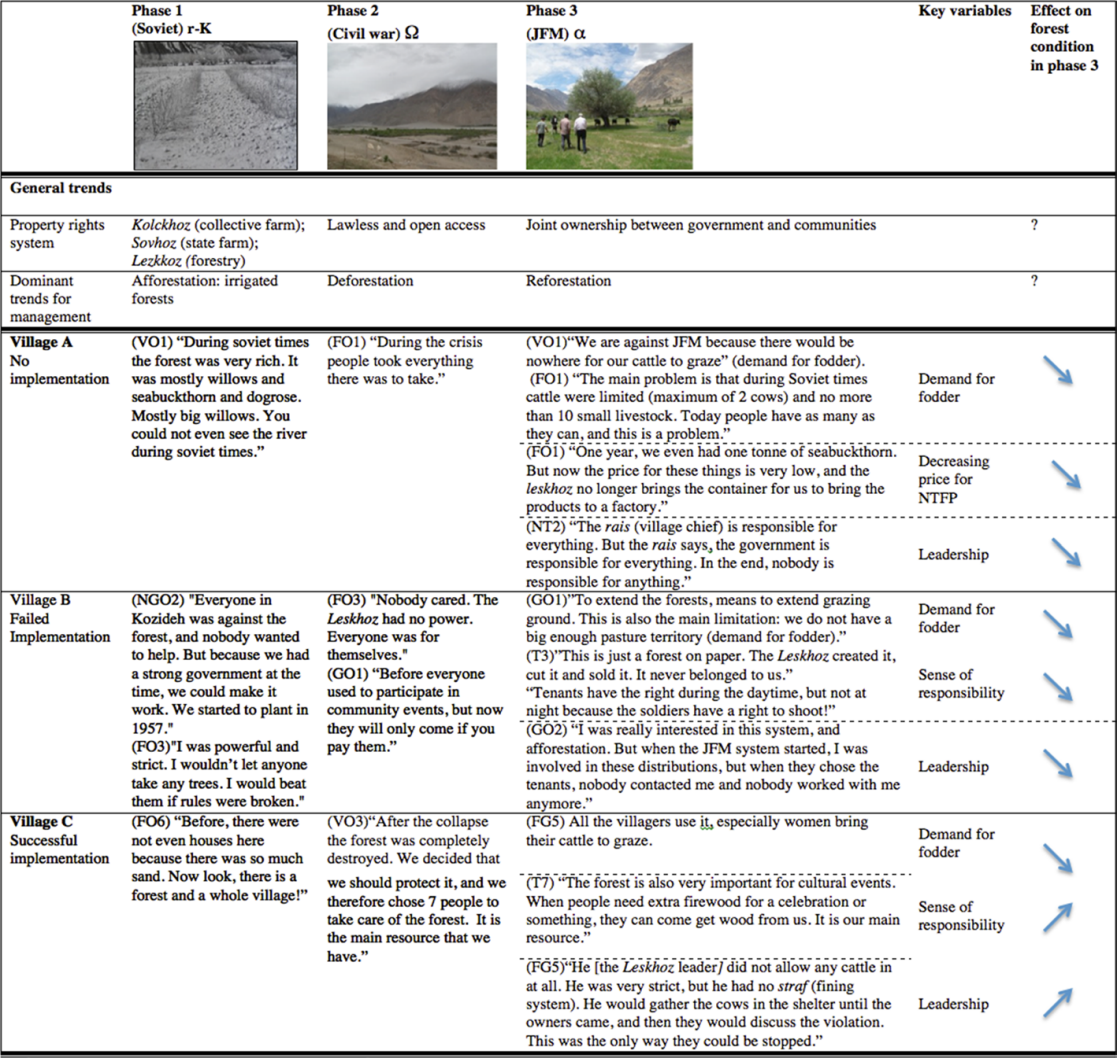
Dominant phases of forestry management—results of qualitative interviews Legend: photograph phase 1: afforestation during Soviet times (1957 in Ptup Ishkashim, photograph by K. Khosimbekov); phase 2: deforested desertified landscape (2012 in Ishkashim, photograph by L.J. Haider); phase 3: *Leskhoz* raid on forest in upper Ishkashim, 2012 (2012 in Ishkashim, photograph by L.J. Haider). Interviewees are coded in brackets (see Appendix for full legend). *VO* village organisation, *FO* forest official, *NGO* NGO, *NT* non-tenant, *T* tenant, *GO* governmental official, *FG* focus group

Three distinct eras emerged from the qualitative interviews: Soviet, civil war and current joint forestry management (Fig. 6). These eras correspond to property rights transitions from state owned, to lawless, to variable collective ownership. The main factors identified as influencing current attitudes towards forest conservation were sense of responsibility, historical leadership and demand for fodder. The way the different communities responded to the shock of the collapse of the Soviet Union and civil war influences current attitudes towards responsibility for the forest, as represented in the quotes in Fig. 6.

## Discussion: context and history matter

Our multi-method approach to understanding the success of JFM in the Pamir Mountains has revealed the importance of historical factors in understanding management success in the present day. We discuss insights from the three approaches: (1) a quantitative analysis of factors enabling collective action guided by the SES framework to explain institutional variability in the success of adoption of joint forestry management in the Pamirs, (2) using Ostrom’s institutional design principles to identify enabling conditions for collective action, and (3) using the adaptive cycle to look at social–ecological dynamics. We discuss insights from each of these in turn identifying and highlighting opportunities for how existing frameworks can be improved, by breaking down the SES framework in contexts where external actors play a role in initiating collective action.

### Variation in success of JFM determined by the structure of the institutional and biophysical environments

Variable history of use, which is an attribute of the users in the SES framework, is important to consider in contexts where management was externally imposed in the past. This result is further supported by the factors regulating tenant density. When *Leskhoz* assigns the number of JFM forest users per forest, they take forest quality into consideration to ensure sufficient revenue potential per forest user. This suggests that the pattern we observed of higher tenant density being related to higher productivity is due to *Leskhoz’s* past decisions, and the absence of any relationship when only Soviet-planted forests are considered. In qualitative interviews, many tenants in the forest with poorer forest condition (village B) complained that many tenants made the management process tedious and not economically viable.

That pre-Soviet forests are positively associated with better forest condition was not unexpected. Not only are they more established forests in better ecological condition, but it is also likely that in the older forests, spiritual and cultural affinity is embedded in local tradition and culture. Our qualitative data suggest that cultural and spiritual affinities are important factors enabling sustainable forest management. As shown in Fig. 6, ‘The forest is also very important for cultural events. When people need extra firewood for a celebration or something, they can come get wood from us. It is our main resource’ (tenant 7). Our results therefore suggest the addition of cultural and spiritual value as additional second-tier variable attributed to the users. Furthermore, breaking ‘history of use’ (U3) down into third- and fourth-tier variables to explicitly include historical leadership and historical monitoring and sanctioning could be important to get a more nuanced picture of reality. Spiritual value could be a fourth-tier variable of ‘importance of resource’ (U8).

### Enabling conditions for collective action

Collective action design principles can be useful in providing a rapid overview of the gaps and strengths in institutions, for both initiating and maintaining collective action (Barnes and Van Laerhoven 2013). We have found that particularly in the case of new participatory management institutions, such as JFM, design principles can help inform the institutional conditions to enable collective action around natural resources. GIZ and *Leskhoz* in Tajikistan have found them to be useful as guiding principles and, based on the recommendations of this study, have since modified their approach to initiating JFM in communities (e.g. in engaging with both present and past key actors, in communicating sanctioning practices more clearly and ultimately devolving more governance responsibility to community-based committees (*personal communication with* GIZ 2014). The document analysis and interviews summarised in Table 2 demonstrate that most design principles for collective action were at least partially met. The JFM programme has been successful in establishing clearly defined boundaries and creating appropriate incentive structures for some communities to encourage best practice (Table 2, design principles 1–2). With regard to design principle 3, only two communities, out of 25, felt they had any authority to influence the rules. This may be due to the legacy of a highly centralised forestry sector and will likely take more than one generation to change. During the Soviet era, the *Leskhoz* managed the forests with no input from communities, which drastically changed under the new era of decentralised joint forest management advocated by non-governmental organisations. While decentralisation has been widely associated with more sustainable forest management, its success is dependent on the ability of forest users to actively participate in the decentralised governance process (Wright et al. 2016; Lund et al. 2018). Explicit attempts at this in Tajikistan include involvement of the communities in monitoring, for example, which only very recently (in 2013) was transferred to forest users. We found in our study that it would be helpful to divide the ‘monitoring’ design principle into two component parts: a) monitoring the users and b) monitoring the resource (sensu Cox et al. 2010). The *Leskhoz* is responsible for monitoring the users but does this only whenever they happen to have a vehicle available to monitor rule breaches (for example, cows grazing on young saplings in the forest). Graduated sanctions exist, and while users know that sanctions have been prescribed, 50% of communities were unaware of what the penalties were and often not aware of what the sanctions were for (for example, grazing vs. extraction). Conflict resolution mechanisms are not in place, and in most communities, conflicts are managed on an ad hoc basis by village leaders. Forest tenants officially hold the right to organise in groups, but only two communities have registered forestry management organisations. Few studies have considered the legacy effects of centralised governance and activities (such as the ones mentioned above) and that these may be barriers to promoting self-organisation in common pool resource studies. Legacy effects on current day leadership are further discussed in the next section.

While the design principles were never meant as a blueprint to be used in the creation of institutions, they outline principles that characterise robust resource management institutions, particularly for common pool resources (Anderies 2004), and as we found in this case can open up a space for dialogue and analysis among users and managers, helping to break down an otherwise seeming overwhelming problem to one that can be tackled systematically. It is important to note that there may be great variation in the specification of design principles across different social and ecological contexts. For example, greater deviation from these principles has been documented to occur in semi-arid ecosystems (such as the Pamirs), due to a more variable environment and in locations where there are greater tensions between formal and informal governance structures (Quinn et al. 2007).

### Understanding reorganisation of forestry management key to understanding current status and future trajectories

Resilience thinking offers a way forwards for institutional analysis to address important historical dynamics (such as historical centralised governance and leadership) that explain institutional variation today by (1) focusing on the dynamics of SES, particularly the reorganisation phase after a major system change, and (2) assessing the effects of slow variables on the capacity of the system to adapt.

As emphasised by Pierson (2004), taking a snapshot of current institutional behaviour may deemphasise the processes through which institutions take shape over time. Understanding historical legacy effects on current conditions is particularly important in places like the Pamirs, where the history of the Soviet Union and its aftermath has left a lasting imprint on the economic, political and social make-up of communities and the ways in which they are governed, Pierson (2004) argues that because social processes are path dependent, and that many social causes and outcomes are slow moving, explaining particular outcomes requires situating them in a temporal sequence of events. Analysis of the reorganisation phase in the Pamirs (starting in ca. 1997 after the civil war ended) demonstrates how historical drivers can influence institutional outcomes in the present. Our interviews (Fig. 6) demonstrate how legacy effects from three distinct institutional phases can strongly determine which variables have the greatest influence on current management systems. The narratives show that leadership qualities and a feeling of responsibility over the forest can be treated as slow variables, which possess path-dependent traits. For example, in village B, a forest tenant explained: ‘This is just a forest on paper. The *Leskhoz* created it, cut it and sold it. It never belonged to us’ (tenant 3). A particularly violent local leader during Soviet times discouraged people to take responsibility of the forest as a communal resource. This attitude remains today, and every household in the village has refused various offers from the *Leskhoz* to take on leadership roles in forestry management, despite payment incentives.

Many of the slow variables such as the ones described above and demographic pressures of grazing shape how planned harvest (as the faster dependent variable) responded to changes in key variables such as property rights that change over time (in this case collapse of the Soviet Union and end of the civil war). For example, the responsibility over forest management felt by community members in village B versus village C could be traced back to how changes in leadership influenced institutional formation over time.

### Reflections on multiple methods and different ways of framing institutional analysis

There exists an inherent tension between the tools and frameworks (e.g. Ostrom 1990, 2009) that are commonly used to analyse institutional dimensions of resource management and the dynamism we know is in inherent to social ecological systems (Anderies 2004). This paper furthers the integration between resilience thinking and institutional analysis (Daedlow et al. 2013). We believe better integrating SES analysis with approaches that take historical processes into account, e.g. through the analysis of slow variables phases of the adaptive cycle (as done here, and see Goulden et al. 2013), or the construction of historical timelines (Resilience Alliance 2010). We offer the following recommendations for scholars undertaking an analysis of the capacity for collective action in natural resource management in cases where there was strong external influence in the past: (1) incorporate explicit historical variables into the SES framework when assessing variation in management success and (2) embed the assessment of fast explanatory variables in the context of slow variables.

The multi-methods approach taken in this study (e.g. Table 2 and Fig. 3) was an attempt to reconcile this tension by examining both the structural elements of the SES of our study system and the dynamics of underlying slow variables. Our experience is similar to that of Basurto et al.’s (2013) in suggesting that the SES framework should be used as a starting, rather than an end point in the study of social–ecological systems. Moreover, we propose that an approach that combines different methods is necessary to help enrich our understanding of the importance of both local contexts and key variables that shape the outcomes of interest over time. We also found that a mixed-method approach can help identify ways in which general frameworks can be tailored to help understand local cases. For example, our findings on the impacts of strong centralised management legacy effects on present-day leadership and self-organisation potential reveal new sub-tier variables to consider such as history of use, leadership and spiritual values. It would be useful to explore the impact of these factors on the success of self-organisation of community natural resource management in other cases to move towards mid-range theory development. However, our study demonstrates the challenge identified by Ban and Cox (2017) that the SES framework lacks user guidance, which results in a plethora of individual research conducted with little coordination, generating problems with establishing causal inference or theory testing across cases (also identified by Lund et al. 2018). Rather, the value of the framework may lie in deep analysis of individual cases studies, to break down the mechanisms of the complex phenomena observed (Ban and Cox 2017), which is certainly the value we found in using the SES framework in our case.

The combination of the quantitative survey with qualitative interviews also allowed us to mitigate limitations of the survey related to low sample size, self-censorship and difficulties of accurate translation that may explain why some key variables that emerged from the qualitative approach were not significant in the quantitative analysis. Many participants may be unwilling to report illegal activity, and respondents may be pressured by NGO staff or government to provided responses in line with how things ‘should’ be. Furthermore, the survey was translated from English to Tajik (the official language of Tajikistan), but some respondents were uncomfortable with Tajik, and therefore, the survey had to be administered in Shugni (a local language) or Russian. This meant that in practice there were often up to four languages spoken during any given focus group, which may have affected clarity of the survey for both the respondents and interviewers. Testing relationships among many social–ecological variables requires a large sample, but collecting large samples is often not possible due to difficulty, cost and the nature of the system being studied (Poteete et al. 2010), such as in the Pamirs where we took an exhaustive sample of all JFM forests, amounting to only 25 cases. Furthermore, large N cross-comparison work is necessarily blind to the nuance of cases that can never be integrated into a meta-analysis. On the other hand, it is important to be aware of the limitations of the qualitative approach: it is difficult to get an overview of relevant themes and issues in the region, and it is difficult to generalise from three cases. Our iterative approach between quantitative and qualitative data collection and analysis allowed us to generalise our results and contribute to theory development, while at the same time maintaining a nuanced perspective in a given place.

## Conclusions

The social–ecological system framework (Ostrom 2009) was useful in exploring why some communities in the Pamir Mountains adopted joint forestry management more successfully than others (with planned harvest as a proxy variable). Tenant density and historical use both helped explain higher planned harvest. Taking a snapshot of a social–ecological system at a particular point in time is not sufficient to explain social or ecological outcomes, particularly in settings characterised by harsh environmental conditions and a strong legacy of centralised management. In such cases, a dynamic approach incorporating slow variables is necessary. Implementers should be aware of the importance of the effects of historical legacy, and institutional analysis may benefit from a more dynamic analysis of the reorganisation phase. Resilience thinking offers a useful set of tools for bringing historical dynamics into social–ecological analysis. We found that differences in forest condition between communities under JFM to be strongly influenced by the historical dynamics of a given place, with a longer history of use, and positive leadership through crisis and reorganisation periods contributing towards more successful outcomes. We call for more research to improve our understanding of how to enable collective action in contexts where there was strong state involvement in resource management in the past (like post-Soviet states). We have much to learn at the interface of institutional scholarship and participatory resource management such as widely promoted by the international development community. Design principles were adopted as a useful framework by the implementing non-governmental organisation and government in designing the expansion of joint forestry management through other parts of Tajikistan as evidenced in the adoption of the Forest Code 2011 (Tajikistan 2011).

## Appendix: 2 Codes for Interviewees

**Table:**

Interview ID	Code	Role	Guiding Questions
*Village A: Gunt valley*			
1. Forest official 1	FO1	Local forest official, monitors and controls use, elder	How has forestry management changed over time? How do you enforce rules? How have violations changed over time?
2. Non-tenant 1	NT1	De facto user (also elder)	What were the forests like before Soviet times? How has land use changed? Do you access the forest? How does the community manage other natural resources?
3. Non-tenant 2	NT2	De facto user (also elder)	As above
4. NTFP woman 1 (transect walk)	NT3	De facto user	How do you use forest resources? How often do you graze cattle here? Where else do you graze your livestock? Would you be able to survive without the forest land?
5. Village chief	VO1	Head of village: in charge of all village systems	What is the distribution of land like? What kind of collection action does the community participate in? (Mills, water, celebration, pasture) How is the community organised? What are the communities assets? Challenges? What are you responsibilities?
6. Plot holder 1	T1	De jure user(non-JFM) On site	How do you use the forest land? When were you given the plot? Is there ever any conflict with non-users? Would you want JFM? Are you aware of the Leskhoz rules? Do you have enough grazing ground?
7. Plot holder 2	T2	De jure user (non-JFM)	As above
8. Focus group forest users (12 users)	FG1	De facto user group	What kind of community institutions exist? How do you use the forest? Do you have enough grazing land? Where do you graze your livestock? What is your main source of livelihood? How do you see the future of your forest? Who is responsible for forestry management? What kind of operational rules exist?
9. GIZ mobiliser for the area	NGO1	Social mobilisers, monitor for the region	What are the main challenges in the region? Why did the community not adopt JFM?
10. District forest official	FO2	Head of Shugnan forestry (Raiyos Koyimnazarov)	Can you explain the Straf process? How were people given land after the collapse of the Soviet Union? History of forest How might the grazing conflicts be resolved? Why do you allow illegal use of forest land?
11. Focus group in neighbouring village	FG2	Neighbouring JFM forest	Is your forest in better or worse condition than neighbouring forests? Is there conflict between users and non-users?
*Village B, Ishkashim valley*			
1. Head of forestry group (tenant 1)	T3	Leader of forest tenants	How do you use the forest land? When were you given the plot? When did you become leader? How were you elected?
2. Head of Jamoat	GO1	Head of the Jamoat (5 villages)	General area characteristics How do the forests in the Jamoat compare? How do you motivate people to act collectively? How easy/hard it is to motivate people? What is the relationship like between villages?
3. GIZ mobiliser for the area	NGO2	Social mobilisers, monitor for the region	What are the opportunities and challenges for this community? Are the tenants cooperative? In your opinion, is this a well or poorly managed forest?
4. Tenant 2	T4	De jure user (JFM)	Do any other collective action organisation exist in the community? Is there a clear leader in the community? What kind of rules did the leader enforce?
5. SUDVO Rais	GO2	Head of para-governmental structure (5 villages)	Do any other collective action organisation exist in the community? Is there a clear leader in the community? What do you see as a solution for more sustainable forestry management?
6. Head of village and VO head	VO2	Head of village (traditional) and head of village (para-governmental)	What are you main responsibilities as head of village? How were you elected? Can you describe how people work together in various management systems? How do you motivate people to act collectively
7. Forest official 1	FO2	Official monitor, fines	How has forestry management changed over time? How do you enforce rules? How have violations changed over time?
8. Focus group with tenants (6)	FG3	De jure user group	Do any other collective action organisation exist in the community? Is there a clear leader in the community? Is there conflict with non-tenants? What is your main challenge?
9. Tenant 3	T5	De jure user (JFM)	As above (T4)
10. Non-tenant 1 (woman)	NT4	De facto user	What were the forests like before Soviet times? How has land use changed? Do you access the forest? How does the community manage other natural resources?
11. Non-tenant 2 (woman + husband)	NT5	De facto user	What were the forests like before Soviet times? How has land use changed? Do you access the forest? How does the community manage other natural resources?
12. Tenant 3	NT6	De jure user (JFM)	As above (T4)
13. Focus group non-tenants (3 women, 2 men)	FG4	De facto user group	Do you use the forest? Do you think the forest is managed well? Fairly? ‘Do you think the way tenants were chosen was fair?
14. Elder 1 (son tenant)	T6	De jure user	How has the village structure changed over time (pre-Soviet, Soviet, post-Soviet?) How did the forest quality, and management change?
*Village C, Ishkashim valley/Wakhan corridor*			
1. Focus group 1	*FG5*	De jure *users* (3 men)	Do any other collective action organisation exist in the community? Is there a clear leader in the community? What kind of rules did the leader enforce?
2. Tenant 1	*T7*	De jure user	When did you become a tenant? What happens if rules are broken? Why do you protect the forest if you are not paid? Before the JFM system, how id you know how much to harvest?
3. Head of village	VO3	Head of village (traditional) and head of village (para-governmental)	What are you main responsibilities as head of village? How were you elected? Can you describe how people work together in various management systems? How do you motivated people to act collectively?
4. Rais Khojagi Dekhoni	GO3	Head of village land committee	What is the distribution of cops Is there enough pasture land? How are the association lands distributed? How do the taxes for the different land categories work?
5. Elder and wife (non-tenants)	NT7	De facto users, elder	How has the village structure changed over time (pre-Soviet, Soviet, post-Soviet?) How did the forest quality, and management change?
6. Former head of village	VO4	De facto user, elder, museum curator, author	What is the history of the two shrines in Namadgut? Why did you decide that the forests were important? How did you motivate people to act collectively?
7. Mirjou (water master)	VO5	Water master; controls distribution of water in the village	What are your main responsibilities? What is the water schedule? What kind of collective action exists around water management? Is there ever conflict around water? How is it resolved?
8. Focus group 2	FG6	Women from tenant households (5)	How often do you go to the forest? What are the main products you use? Do any informal rules on grazing exist? Why is the forest important to you?
9. Focus group 3	FG7	Non-tenants women and men(8)	Do you use the forest? Do you think the forest is managed well? Fairly? Do you think the way tenants were chosen was fair?
10. Forest leader	T8	(Transect walk)	What are the main uses of your plot? Why were you chosen as forest leader? How do you enforce rules?
11. Tenant 2	T9	Woman (transect walk)	- What are the main uses of your plot? - Do you use NTFP? - Why, as a woman, did you want to become a forest tenant? Do you face particular difficulties?
	*Khorog*		
1. Retired forest official	FO3	Head of forestry, retired	How has forest management changed over time? Why did you start foresting Ishkashim area? How did *Leskhoz* adjust to changes in government?
2. Regional government official 1	FO4	Land management Shugnan	What is the distribution of pasture in the district? How much agricultural land? How much pasture land? Land conflict?
3. MSDSP 1	NGO3	NRM official	How many villages and jamoats selected forestry as a management priority? Do any saving’s groups use funds for forestry management or other natural resource management?
4. MSDSP 2	NGO4	Civil Society official	How does your community saving’s group work?
5. Forest Official	FO4	Head of land-use specification	What were the main goals in afforesting Ishkashim valley? What are the main changes that occurred since the 1970 s?
